# Isolation and characterization of *Magnetospirillum* sp. strain 15-1 as a representative anaerobic toluene-degrader from a constructed wetland model

**DOI:** 10.1371/journal.pone.0174750

**Published:** 2017-04-03

**Authors:** Ingrid Meyer-Cifuentes, Paula M. Martinez-Lavanchy, Vianey Marin-Cevada, Stefanie Böhnke, Hauke Harms, Jochen A. Müller, Hermann J. Heipieper

**Affiliations:** 1 Helmholtz Centre for Environmental Research—UFZ, Department of Environmental Biotechnology, Leipzig, Germany; 2 Technical University of Denmark, Bibliometrics and Data Management, Department for Innovation and Sector Services, Lyngby, Denmark; 3 Helmholtz Centre for Environmental Research—UFZ, Department of Environmental Microbiology, Leipzig, Germany; University of Münster, GERMANY

## Abstract

Previously, Planted Fixed-Bed Reactors (PFRs) have been used to investigate microbial toluene removal in the rhizosphere of constructed wetlands. Aerobic toluene degradation was predominant in these model systems although bulk redox conditions were hypoxic to anoxic. However, culture-independent approaches indicated also that microbes capable of anaerobic toluene degradation were abundant. Therefore, we aimed at isolating anaerobic-toluene degraders from one of these PFRs. From the obtained colonies which consisted of spirilli-shaped bacteria, a strain designated 15–1 was selected for further investigations. Analysis of its 16S rRNA gene revealed greatest similarity (99%) with toluene-degrading *Magnetospirillum* sp. TS-6. Isolate 15–1 grew with up to 0.5 mM of toluene under nitrate-reducing conditions. Cells reacted to higher concentrations of toluene by an increase in the degree of saturation of their membrane fatty acids. Strain 15–1 contained key genes for the anaerobic degradation of toluene via benzylsuccinate and subsequently the benzoyl-CoA pathway, namely *bssA*, encoding for the alpha subunit of benzylsuccinate synthase, *bcrC* for subunit C of benzoyl-CoA reductase and *bamA* for 6-oxocyclohex-1-ene-1-carbonyl-CoA hydrolase. Finally, most members of a clone library of *bssA* generated from the PFR had highest similarity to *bssA* from strain 15–1. Our study provides insights about the physiological capacities of a strain of *Magnetospirillum* isolated from a planted system where active rhizoremediation of toluene is taking place.

## Introduction

Rhizoremediation is a process in which most degradation of toxic compounds is attributed to the activity of microbes in the rhizosphere [1]. This process has the capacity to attenuate pollutant concentrations in contaminated environments quite efficiently. Microbial metabolic activity, community establishment, and proliferation in the rhizosphere are augmented by the release of root exudates and oxygen by plants. In addition, roots modify the chemical and physical properties of soils and thus enhance the availability of pollutants for microbes [1, 2]. Common pollutants found in soil are benzene, toluene, ethylbenzene and xylenes (collectively known as BTEX), which are all products of the petroleum industry. Microbial toluene transformation has been extensively studied and several aerobic and one anaerobic catabolic pathways are known [3–7]. Aerobically, various oxygenases introduce hydroxyl groups and cleave the aromatic ring while anaerobically, toluene is converted to benzylsuccinate via the addition of its methyl group to fumarate. The latter step is catalyzed by highly oxygen sensitive benzylsuccinate synthase (BssABC) [8]. Benzylsuccinate is further transformed via a β-oxidation-like scheme to benzoyl-CoA, a central intermediate in the anaerobic degradation of many aromatic compounds [9]. Benzoyl-CoA is then converted in a two-electron reduction to cyclohexa-1,5-diene-1-carbonyl-CoA [10] or by an apparent four-electron reduction to cyclohex-3-ene-1-carboxylate-CoA in *Rhodopseudomonas palustris* [11]. In facultative anaerobes the reduction is performed by ATP-dependent Class I benzoyl-CoA reductase (BcrABCD) whereas obligate anaerobes employ an ATP-independent Class II reductase (probably BamBCDEFGHI). The 6-carbon atom ring is eventually cleaved by consecutive water-addition, dehydrogenation and hydrolytic ring fission catalyzed by 6-oxocyclohex-1-ene-1-carbonyl-CoA hydrolase (BamA) [6]. Further degradation yields acetyl-CoA units [12].

To study microbial transformations in the rhizosphere under controlled conditions, a laboratory-scale planted fixed-bed reactor (PFR) was designed. Within this PFR, a circulating flow regime prevents the formation of large chemical gradients and an efficient turnover of organic carbon occurs [13]. In previous studies on a PFR running for several years with toluene as sole external carbon source, it was shown that toluene was mainly degraded aerobically by members of the *Burkholderiales* [14, 15]. However, several cultivation-independent analyses suggested that microbes able to degrade toluene anaerobically were also plentiful. DNA microarray analysis revealed the presence of the *bssA* gene, coding for the alpha subunit of benzylsuccinate synthase, most similar to the one of *Magnetospirillum* sp. TS-6 [15, 16], an α-proteobacterial denitrifying toluene degrader [17]. Based on 16S rRNA gene amplicon sequencing, microbes of this genus comprised up to 6% of the total bacterial community in the PFR. Most of those sequences had 100% identity with the respective 16S rRNA gene segments of *Magnetospirillum* sp. TS-6 [16]. Comparing relative abundance of *Magnetospirillum* 16S rRNA gene amplicons with qPCR enumerations of the *bssA* and 16S rRNA genes indicated that many Magnetospirilli in the PFR carried *bssA* [15]. Reverse Transcription-qPCR analysis showed that *bssA* transcripts were highly abundant [14]. Yet although *bssA* of apparent *Magnetospirillum*-origin was highly transcribed in the PFR, corresponding peptides were not detected by comprehensive metaproteomics [14]. In contrast, various other proteins detected in the PFR, including benzoyl-CoA reductase, were phylogenetically assigned to *Magnetospirillum*, suggesting that benzylsuccinate synthase was indeed absent rather than present but not detected.

The presence of high numbers of microbes capable of degrading toluene anaerobically in this PFR is not yet understood. On the basis of these previous observations, we aimed at isolating bacteria able to degrade toluene under nitrate-reducing conditions from a PFR. We obtained a strain of *Magnetospirillum* sp. which was further characterized from a physiological and genetic point of view to obtain an insight into their possible role in the PFRs. Additionally, their tolerance mechanism to toluene was studied in order to elucidate their potential to thrive in these wetland models.

## Materials and methods

### Growth medium

Enrichment, isolation and cultivation of strain 15–1 were carried out in a mineral medium previously described by Tschech and Fuchs [18]. Toluene (0.3 mM—0.5 mM) was supplied to liquid medium and acetate (5 mM) to solid medium as the sole carbon source. KNO_3_ (5 mM) was added as electron acceptor, Na_2_S (0.25 mM) as reducing agent and resazurin (0.8 μM) as redox indicator. The pH was adjusted to 7.1 with 1M NaOH. For anaerobic cultivation, 50 mL of medium was flushed in 120 mL-serum bottles with N_2_ for 25 min. The bottles were then sealed with Teflon-coated rubber stoppers and crimps and subsequently autoclaved. Vitamins, FeSO_4_, Mg/Ca [17] and Na_2_S solutions were added subsequently from sterile stock solutions with N_2_-flushed syringes. Prior to inoculation, the medium was shaken at room temperature until reduction of resazurin. For aerobic cultivation, KNO_3_ and Na_2_S were omitted and free gaseous exchange with the ambient atmosphere was permitted under sterile conditions.

### Enrichment, isolation and characterization

Samples of gravel and roots (1 gram) were taken from the PFR that had been planted with *Juncus effusus* and run continuously with up to 0.5 mM of toluene as sole external carbon source for several years [16]. The samples were washed with sterile phosphate buffer (10 mM, pH 7.1) and placed in triplicate in 120-mL serum bottles containing anoxic mineral media, supplied with 0.37 mM of toluene and 5 mM of potassium nitrate. Non-inoculated (negative) controls were set up during the whole isolation process in order to determine whether toluene losses observed in the inoculated bottles were due to abiotic effects. Toluene concentration was measured in the inoculated bottles and negative controls every 72 h. Additional toluene was injected to the microcosms when the substrate was completely degraded. Turbid cultures were diluted serially and the maximally diluted culture was further transferred to new media. Pure cultures were obtained by repeatedly plating anaerobically on agar with acetate as the carbon source and verifying purity microscopically.

### Growth substrates

The isolated strain was tested for growth with different aromatic and non-aromatic compounds as sole carbon and energy source under nitrate-reducing and oxic conditions (Table 1). Cultures growing exponentially with toluene as sole carbon source were used as pre-inoculum for growth tests with aromatic compounds. Inocula pre-cultured with sodium succinate were used for testing non-aromatic compounds. Cultivations were performed in duplicate at 30°C, and bacterial growth was assessed by measuring optical density with a spectrophotometer (UV/VIS Spectrometer Lambda 2S, Perkin-Elmer, USA) at 560 nm and by cell counting with a Coulter Counter® (Beckman Coulter Inc), software Multisizer 3 Version 3.51® (Beckman Coulter Inc.). Growth with the tested substrates was defined as at least a doubling in the optical density.

**Table 1:** Anaerobic and aerobic growth tests of denitrifying strain 15–1 with different aromatic and non-aromatic compounds as sole carbon sources.

Aromatic	Conc.	Aerobic	Anaerobic	Non-aromatic	Conc.	Aerobic	Anaerobic
compounds	(mM)	Growth	Growth	compounds	(mM)	Growth	Growth
toluene	0.5	-	+	acetate	5	+	+
*m*-xylene	0.05	-	-	pyruvate	5	+	+
*o*-xylene	0.05	-	-	fumarate	1	+	+
*p*-xylene	0.05	-	-	succinate	5	+	+
*m*-cresol	0.7	-	+	ethanol	5	+	-
*o*-cresol	0.5	-	-	glucose	5	NT	-
*p*-cresol	0.5	-	+	fructose	5	NT	-
benzoate	5	+	+	galactose	5	NT	-
2-hydroxybenzoate	1	-	-	sucrose	5	NT	-
3-hydroxybenzoate	0.7	-	-				
4-hydroxybenzoate	0.7	+	-				
2-aminobenzoate	0.7	-	-				
3-aminobenzoate	0.7	-	-				
4-aminobenzoate	0.7	-	-				
phenylacetate	1	-	-				
Monochlorobenzene	0.05	-	-				

+, growth; -, no growth; NT, not tested

### Toxicity test for toluene

In order to test the toxic effect that toluene has on isolate 15–1 during growth with a readily metabolisable carbon source (such as succinate), toluene was added at different concentrations to exponentially growing cultures as described by Heipiper *et al*. [19]. The control was a culture growing only with succinate as the carbon source. All experiments were performed in mineral media under anoxic conditions at 30°C.

Growth inhibition after addition of toluene was measured in percentage in relation to the control by comparing the differences in growth rate μ (h^−1^) between intoxicated cultures (μ_toxin_) with that of control cultures (μ_control_). From the toxicity test, other parameters such as the half-maximal effective concentration **(**EC50) and minimum inhibitory concentration parameter [20] were also determined.

### Fatty acid analyses

Cells growing with succinate and exposed to different concentrations of toluene (toxin) were harvested by centrifugation after 35 hours in the presence of the toxic agent and washed with phosphate-buffer (50 mM, pH 7.0). Lipids were extracted with chloroform/methanol/water as described by Bligh and Dyer [21]. Fatty acid methyl esters (FAME) were prepared by 15 min incubation at 95°C in boron trifluoride/methanol using the method of Morrison and Smith [22]. FAME were extracted with hexane and analyzed using a GC-FID System (HP5890, Hewlett & Packard, USA) and a CP-Sil 88 capillary column (Chrompack, Middelburg, The Netherlands; length, 50 m; inner diameter, 0.25 mm; 0.25-μm film). The various types of fatty acids were identified with the aid of standards. The relative amounts of the fatty acids were determined from the peak areas of the FAME.

### 16S rRNA gene sequence analysis

The 16S ribosomal RNA gene was amplified from genomic DNA extracted with DNeasy Blood and Tissue Kit (Qiagen, Germany). Amplifications were performed by PCR in triplicate using the universal primers 27F and 1492R [23]. 16S rRNA gene amplicons were purified with a QIAquick PCR purification Kit (Qiagen, Germany) according to the manufacturer’s instruction. Sanger sequencing of the PCR products from both ends was outsourced (GATC Biotech AG, Cologne, Germany).

A phylogenetic tree of the partial 16S rRNA gene sequence of the isolate and those of reference sequences was constructed by using MEGA version 7 [24]. The evolutionary history was inferred using the Neighbor-Joining method [25]. The evolutionary distances were computed using the Maximum Composite Likelihood method and are in the units of the number of base substitutions per site. The analysis was repeated on 1000 bootstrap samples to obtain confidence estimates of the branching order [26].

### Catabolic genes amplification and sequencing

The genes *bcrC*, *bamA* and *bssA* were amplified through PCR with the respective primers shown in S1 Table using DNA from the isolate as template. Genomic DNA was obtained as mentioned above. PCR conditions for the primer sets were: 15 min at 95°C and 35 cycles of 30 s at 95°C; 30 s at 54°C (*bamA*), or 70°C (*bcrC*); 1 min at 72°C; and 10 min at 72°C. For *bssA* the amplification program was: 15 min at 95°C and 35 cycles of 45 s at 94°C; 45 s at 56°C; 2 min at 72°C; and 8 min at 72°C. The expected PCR products sizes were 1546, 800, and 800 bp for *bssA*, *bcrC* and *bamA*, respectively.

PCR products were purified with a QIAquick PCR purification Kit (Qiagen, Germany) according to the manufacturer’s instruction. PCR products were sequenced for both strands at GATC Biotech AG, (Cologne, Germany). Assembly of forward and reverse reads was carried out by using the program Geneious version 8 [http://www.geneious.com, [27]] and homology searches were performed through BLAST (http://www.ncbi.nlm.nih.gov/).

### Clone library of *bssA* from the PFR

Pore water samples (180 ml in total) were collected from the PFR with a syringe, filtered, and DNA was extracted as described by Martinez-Lavanchy *et al*. [16]. The *bssA* gene was amplified with bssAF1-bssAR1 (S1 Table) primers (0.5 μM) using the PCR program described above. The obtained PCR products were purified using ZymocleanTM DNA Recovery Kit (Zymo Research) according to manufacturer instructions and ligated into pGEM-T-Easy vector (Promega). Ligation and transformation of competent cells of *Escherichia coli* JM109 was carried out according to the manufacturer’s protocol and as described in Sambrook and Russell [28]. To screen for clones with correct inserts, colony PCR was carried out as detailed in Woodman [29] using primers M13-F and M13-R (S1 Table). The PCR program used was 95°C for 3 min, 30 cycles at 94°C for 40 sec, 52°C for 40 sec and 72°C for 1 min, and a final elongation at 72°C for 10 min. Ninety six PCR fragments of the correct size were purified and partially sequenced using the M13-F primer at GATC Biotech AG (Cologne, Germany).

The obtained *bssA* sequences were translated and the resulting amino acid sequences aligned to several reference sequences obtained from GenBank (http://www.ncbi.nlm.nih.gov/) and the one of isolate 15–1. All translations were carried out by using the program Geneious version 8. Phylogenetic and molecular evolutionary analyses were conducted using MEGA version 7. The evolutionary history was inferred using the Neighbor-Joining method and evolutionary distances were computed using the Poisson model. The analysis was repeated on 1000 bootstrap samples to obtain confidence estimates of the branching order.

### Chemical analysis

Toluene concentration was measured using a GC-FID (Agilent HP7890, USA) equipped with a 30 m x 320 μm x 5 μm film thickness Agilent DB-MTBE column. The analytical setup was according to Martinez-Lavanchy *et al*. [30].

Nitrate (NO_3_^-^) and nitrite (NO_2_^-^) concentrations were measured using ion chromatography (DX 500, Dionex, USA). The column used for the analysis was an IonPac AG4A-SC (4x50 mm and AS4A-SC 4x2501).

### Nucleotide sequence accession number

The partial 16S rRNA gene sequence and sequences of the *bssA*, *bcrC* and *bamA* gene fragments of the isolated strain have been deposited in GenBank under the accession numbers KX373668, KX815328, KX815329 and KX815330 respectively.

## Results

### Bacterial isolation

**Fig 1:**
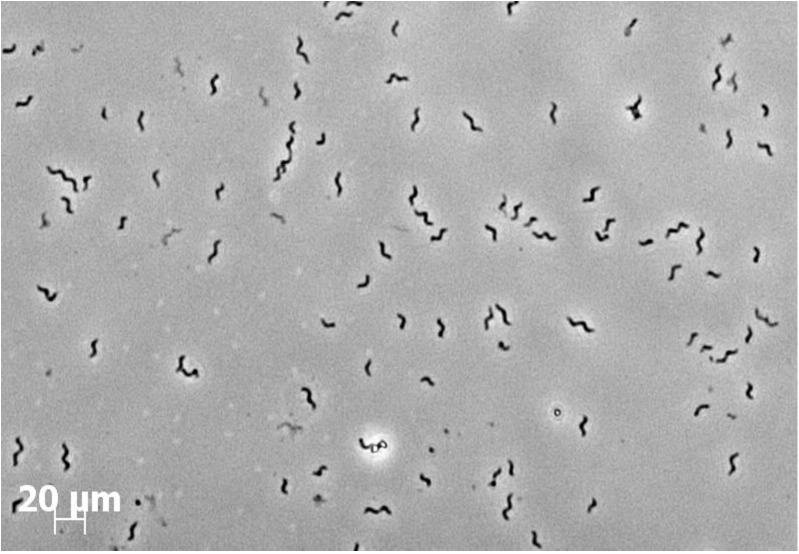
Microscopy observation of strain 15–1 isolated from a Planted Fixed-bed Reactor (PFR).

Microcosms were subjected to several transfers until bacterial growth and toluene utilization was observed to be stable. Consecutively, turbid cultures were serially diluted from 10^−1^ to 10^−5^. After 40 days of incubation, the highest dilution showing turbidity and toluene degradation was the dilution 10^−3^. Aliquots of this dilution were transferred in triplicate bottles with fresh anoxic medium and 0.5 mM of toluene was supplied. After several transfers a spiral-shaped bacterium was observed microscopically to be predominating in these cultures. Finally, colonies of circular, convex, mucoid and opaque were isolated from acetate-agar plates after 2 weeks of anoxic incubation. Microscopically, the isolated colonies consisted of a spiraled, 3–7 μm long and motile bacterium (Fig 1). Motility was not attributed to magnetotaxis since the bacteria was unable to respond to a magnetic field (data not shown). One isolate was designated strain 15–1 and used in all further investigations.

**Fig 2:**
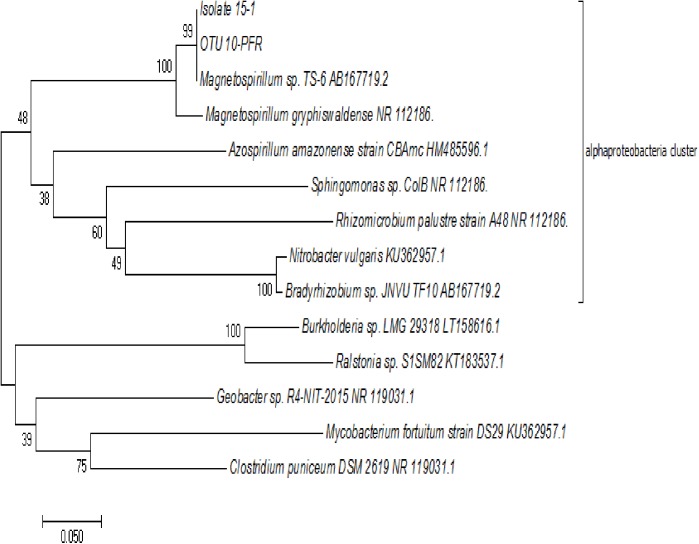
Phylogenetic tree of 16S rRNA gene sequences. The isolated strain obtained in this study is designated 15–1.

Phylogenetic analysis of the partial 16S rRNA gene showed that strain 15–1 was most closely related to *Magnetospirillum* sp. TS-6 among cultured microbes (99% sequence identity). Those Illumina 16S rRNA gene amplicons obtained previously from the PFRs that affiliated with *Magnetospirillum* (OTU 10-PFR-2; 6% relative abundance of all OTUs) had 100% identity with the respective 16S rRNA amplified region of strain 15–1 (Fig 2).

### Growth with toluene under nitrate-reducing conditions

**Fig 3:**
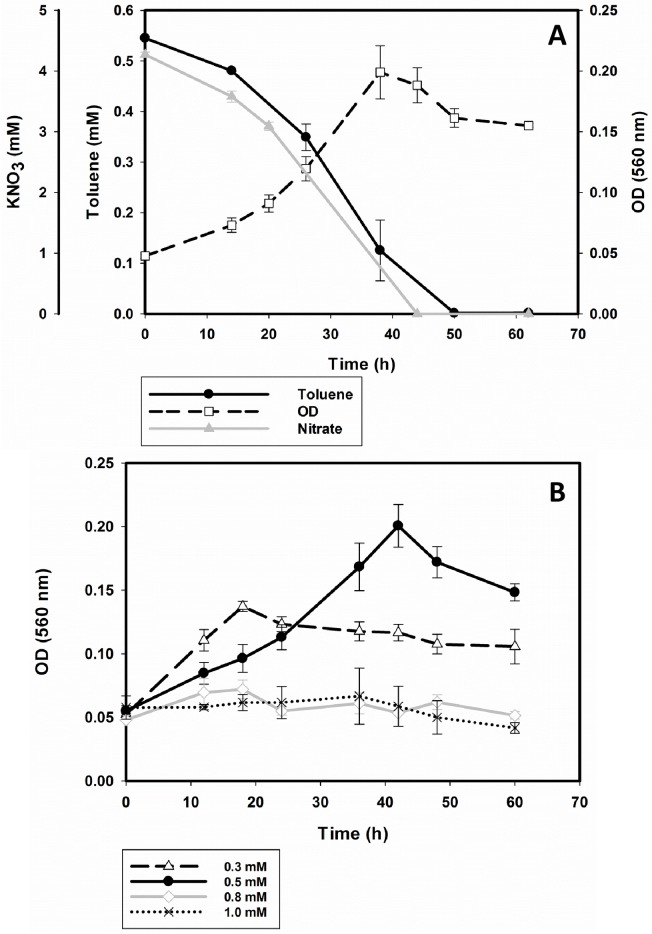
Anaerobic growth of strain 15–1 with toluene under nitrate reducing conditions with 0.5 mM of toluene (A) and different concentrations of toluene (B) as sole carbon and energy source.

Cells of strain 15–1 were cultured anaerobically in mineral medium using toluene at different concentrations (0.3 to 1 mM) as sole energy and carbon source and 5 mM of KNO_3_ as terminal electron acceptor. Under these conditions, strain 15–1 was able to degrade 0.5 mM of toluene within 50 hours (Fig 3A). Toluene concentrations of 0.8 mM and higher did not support growth (Fig 3B). Overall, degradation of toluene was attributed to bacterial activity, since insignificant losses were observed in negative controls (data not shown). The initial velocities of degradation were calculated from the kinetics of the 0.3 mM and 0.5 mM experiments by considering the 3–4 first points of the curves. Both data sets were fit to a polynomial curve and the derivative dC/dt was calculated in order to obtain the rate of the reaction. Both data sets presented a first order kinetics with an average rate constant of 0.044±0.004 (1/hr).

The highest biomass was achieved in cultures growing with 0.5 mM of toluene. The maximum optical density was 0.21 which corresponded to 2·10^8^ cells/ml. The growth rate (μ) varied depending on the initial concentration of toluene in the medium. With 0.3 mM of toluene μ was about 0.01 h^-1^ while with 0.5 mM of toluene μ increased to about 0.04 h^-1^ which corresponds to a doubling time of approximately 17 hours. The terminal electron acceptor NO_3_^-^ was depleted concomitantly with toluene (Fig 3A). Nitrite was never detected during any cultivation of strain 15–1.

### Growth of strain 15–1 with other substrates

The growth of strain 15–1 with different aromatic and non-aromatic compounds using mineral medium was tested under nitrate-reducing and oxic conditions. Only a few aromatic compounds such as cresols supported growth under both conditions (Table 1). On the other hand, toluene supported bacterial growth exclusively under anoxic conditions.

In growth experiments using non-aromatic compounds, the isolate grew well with all organic acids tested under both oxic and anoxic conditions (Table 1).

### Detection of catabolic genes in strain 15–1

The partial genes for benzylsuccinate synthase, subunit A (*bssA*), benzoyl-CoA reductase, subunit C (*bcrC*) and 6-oxocyclohex-1-ene-1-carbonyl-coenzyme A hydrolases (*bamA*) were successfully amplified from cultures of strain 15–1 with fragment sizes of 1546, 800 and 800 bp respectively (S1 Fig). BLAST analysis showed that *bssA* and *bcrC* had 99% and 98% identity respectively with the corresponding genes from *Magnetospirillum* sp. TS-6 described previously by Shinoda *et al*. [17]. The partial *bamA* gene had 98% identity with the one from *Magnetospirillum* pMbN1 described previously by Lahme *et al*. [31].

### Phylogenetic analysis of BssA sequences from PFR-clones

**Fig 4:**
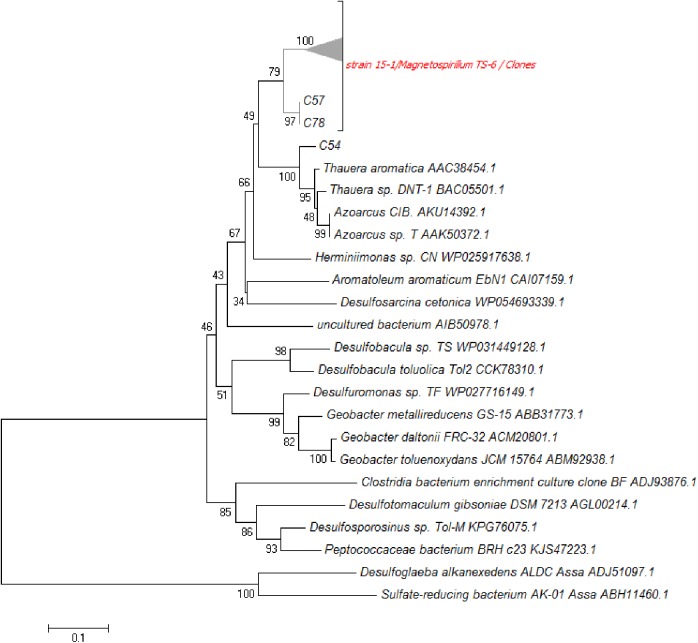
Neighbor joining tree of BssA amino acidic sequences. The cluster shown as a triangle represents members of a clone library obtained from a PFR closely related to *Magnetospirillum* strain TS-6 and to the isolate 15–1. Clones 57 and 78 are part of a second subgroup and C54 is shown in the *Thauera* cluster

Partial *bssA* was PCR amplified from PFR pore water samples, a clone library was prepared from these amplicons and a phylogenetic tree for BssA amino acid sequences was constructed (Fig 4). From 91 sequenced clones, 88 (96.7%) were most similar to *bssA* sequences from strain 15–1 and *Magnetospirillum* sp. TS-6. The nucleotide sequences of these 88 sequences showed 93 to 99% similarity to the genes from *Magnetospirillum* sp. TS-6 and strain 15–1 and computed amino acid sequences were 99–100% similar to benzylsuccinate synthase from both strains. Two clone-sequences, C57 and C78, had only 92% similarity to the sequence from strain 15–1 and formed a second subgroup in the phylogenetic tree. The amino acid sequence from clone C54 was most closely related (95%) to BssA of *Thauera aromatica*. Alignment with the remaining two clone sequences, C26 and C92, revealed high dissimilarities to BssA and considered amplification artifacts; therefore they were excluded in this study.

### Toxicity of toluene to strain 15–1

**Fig 5:**
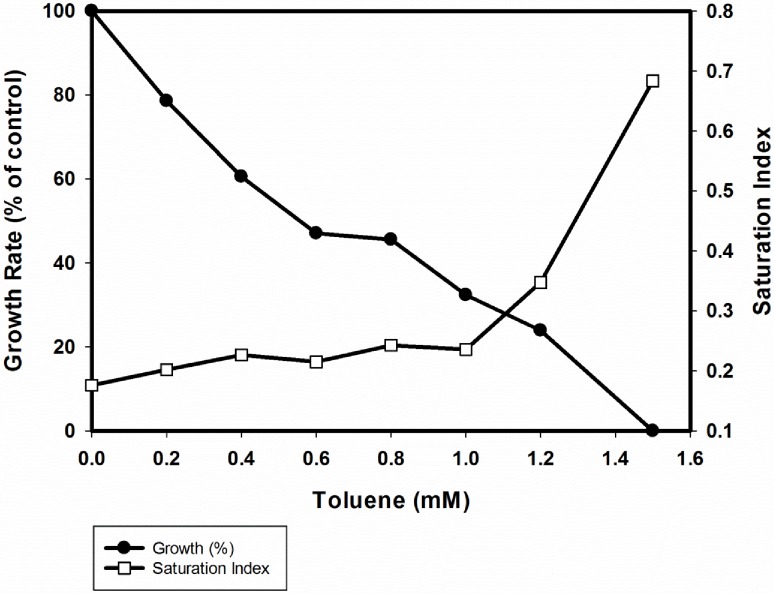
Effect of toluene on growth (closed circles) and degree of saturation of membrane fatty acids (open squares) of strain 15–1.

In order to elucidate the tolerance of the new isolate towards toluene, we carried out growth inhibition tests. Toluene was added as a toxin in different concentrations to cells growing with succinate at the beginning of growth. Subsequently, optical density was measured and relative growth rates were calculated in relation to the control (cultures without the toxin). Finally, growth rates were plotted against the different concentrations of the toxin tested (Fig 5). The effective concentration (EC50) of toluene leading to 50% growth inhibition and the minimal inhibitory concentration (MIC) of toluene were calculated and corresponded to 0.55 and 1.5 mM, respectively. With 1.5 mM of toluene, bacterial growth was immediately inhibited after addition of the toxin (Fig 5).

### Membrane fatty acid composition

**Fig 6:**
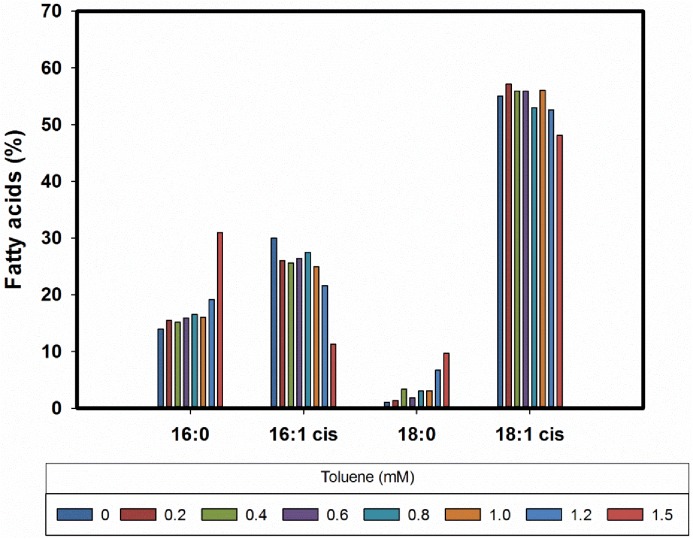
Effect of toluene on the membrane fatty acids pattern of strain 15–1.

Predominant fatty acids of *Magnetospirillum* strain 15–1 were palmitic acid (C16:0), palmitoleic acid (C16:1*cis*), stearic acid (C18:0), and *cis*-vaccenic acid (C18:1Δ11*cis*) which together constituted more than 95% of the fatty acid content of the strain. In the presence of toluene, the amount of the unsaturated fatty acids (C16:1 and C18:1) decreased with a subsequent increase in the amount of the saturated fatty acids (C16:0 and C18:0) (Fig 6). In addition, Fig 5 shows the effect of toluene on the degree of saturation of fatty acids as major sum parameter expressing the rigidity of the membrane [17, 31–36]. A drastic increase of the saturation index was observed only at toluene concentrations above 1.0 mM.

## Discussion

This work shows that the isolated strain 15–1 belongs to the genus *Magnetospirillum*. Several members of the genus *Magnetospirillum* have been described such as *M*. *aberrantis*, *M*. *gryphiswaldense*, *M*. *bellicus*, *M*. *magnetotacticum* MS-1, *M*. *magneticum* AMB-1, strains pMbN1 and TS-6 [17, 31–36]. All these microbes can grow with various carbon sources, either aerobically or anaerobically by nitrate reduction as observed for the isolate 15–1. However, only few strains of this genus have been identified as aromatic compound degraders [17, 31].

Apparently, *Magnetospirillum* sp. 15–1 is one of the few strains within the anaerobic toluene degraders that can degrade up to 0.5 mM of toluene completely and can tolerate elevated concentrations of the compound when growing with succinate [37, 38]. Similarly to other strains of *Magnetospirillum*, strain 15–1 grew well with various organic acids and sugars were not utilized [32, 35, 39]. Strain 15–1 grew only with a few aromatic compounds such as toluene, 4-hydroxybenzoate, *p*- and *m*-cresol; its substrate range is thus more restricted than that reported for *Magnetospirillum* sp. TS-6 [17].

Based on previous reports regarding catabolic pathways for toluene degradation [40, 41], we propose that *Magnetospirillum* strain 15–1 degrades toluene anaerobically via the benzylsuccinate pathway. We confirmed the presence of key genes involved in the proposed pathway after DNA amplification. Therefore, strain 15–1 resembles strain TS-6 in that it most possibly metabolizes toluene under denitrifying conditions [17].

BssA sequence analysis of the isolate 15–1 and PFR content revealed that the dominant microbe capable of anaerobic toluene degradation in these systems belongs to *Magnetospirillum* genus although toluene was apparently not used by these microbes in the reactor at the time. Additionally, one *bssA* sequence most similar to those of *Thauera aromatica* was obtained even though no respective 16S rRNA gene amplicon was detected.

Regarding toxicity, toluene and other hydrophobic molecules are known to accumulate in membranes causing disturbance of structure and function [42, 43]. In this study, a slight variation on the composition of saturated fatty acids as a possible response to an increment in the membrane fluidity was detected. As reported for cells of *T*. *aromatica* and *Geobacter sulfurreducens* we observed an increase of saturated fatty acids and a relative decrease in unsaturated fatty acids upon exposure to selected organic compounds. The highest response in the degree of saturation occurred at 75% of cell inhibition which is in agreement with previous data reported by Duldhardt *et al*. [44]. Tolerance of *Magnetospirillum* sp. 15–1 towards toluene was shown to be within the range reported for other anaerobic bacteria such as *T*. *aromatica* and *G*. *sulfurreducens* [45]. It has been suggested that *Magnetospirillum* strains can tolerate toluene by exporting toxic levels from the cytosol by using Bcr/CF1A drug resistance transporters encoded by the *bssH* gene contained in the *bss* operon [40, 46].

Based on our findings, we suggest that *Magnetospirillum* sp. 15–1 and similar strains can function in the PFR as anaerobe toluene-degraders, albeit restricted to conditions appropriate for the synthesis of benzylsuccinate synthase. During the 6-year long operation of the PFR there were several time frames during which the pore water was anoxic and the redox potential below –300 mV. It is conceivable that *Magnetospirillum* strains catabolized toluene anaerobically during those times. The magnitude of anaerobic toluene degradation was likely lower than that observed for aerobic degradation; the toluene degradation rate of PFR with about 10^6^ bacterial cells/ml (6% of which were *Magnetospirillum*) was 40% faster than that of batch cultures of strain 15–1 with 2·10^8^ cells/ml [14, 15].

Under conditions unsuitable for the synthesis of benzylsuccinate synthase, *Magnetospirillum* strains may mainly thrive on organic acids as part of and derived from root exudates [47, 48]. Lu *et al*. found *Magnetospirillum* to be highly abundant in the rice rhizosphere, possibly degrading products of root exudates [49]. The ability of *Magnetospirillum* strains to thrive at the oxic/anoxic interface in natural ecosystems [50] together with their catabolic capabilities could allow *Magnetospirillum* strains to be present and survive for a long period in environments such as the PFRs. Further studies in the constructed wetlands model systems are necessary to elucidate the physical and chemical conditions (e.g. O_2_, partial pressure and nitrate concentration) which are affecting synthesis of benzylsuccinate synthase from *Magnetospirillum*.

## Supporting information

S1 Fig Catabolic genes amplification using genomic DNA of 15–1 strain growing on toluene as the sole carbon source. Lanes A, B and C shows *bssA*, *bamA* and *bcrC* amplicons respectively. Lanes D,E and F positive controls for each gene in the same order. Lanes G, H and I, negative controls of each sample.
(DOCX)

S1 Table Primers used for amplification of 16S rRNA and catabolic genes. (DOCX)
