# Supplementary material for: Isolation and characterization of Magnetospirillum sp. strain 15-1 as a representative anaerobic toluene-degrader from a constructed wetland model
Source: PLoS One. 2017 Apr 3;12(4):e0174750. doi: 10.1371/journal.pone.0174750 (PMC5378359; doi:10.1371/journal.pone.0174750)
Supplement: S1 Fig — Lanes A, B and C shows bssA, bamA and bcrC amplicons respectively. Lanes D,E and F positive controls for each gene in the same order. Lanes G, H and I, negative controls of each sample. (DOCX) [file pone.0174750.s001.docx]

**
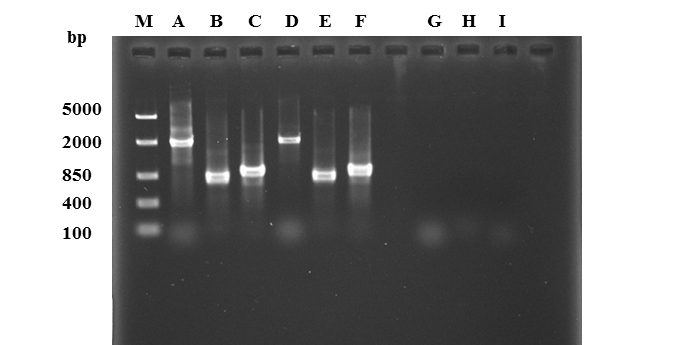
Figure S1.** Catabolic genes amplification using genomic DNA of 15-1 strain growing on toluene as the sole carbon source. Lanes A, B and C shows bssA, bamA and bcrC amplicons respectively. Lanes D,E and F positive controls for each gene in the same order. Lanes G, H and I, negative controls of each sample.
