# Supplementary material for: Isolation and characterization of Magnetospirillum sp. strain 15-1 as a representative anaerobic toluene-degrader from a constructed wetland model
Source: PLoS One. 2017 Apr 3;12(4):e0174750. doi: 10.1371/journal.pone.0174750 (PMC5378359; doi:10.1371/journal.pone.0174750)
Supplement: S1 Table — (DOCX) [file pone.0174750.s002.docx]

**Table S1.** Primers used for amplification of 16S rRNA and catabolic genes.

| **Primers** | **Target gene** | **Sequence** | **Reference** |
| --- | --- | --- | --- |
| **27F** | ***16S* rRNA** | **AGAGTTTGATCMTGGCTCAG** | **Weisburg et al., 1991** |
| **1492R** | ***16S* rRNA** | **ACCTTGTTACGACTT** | **Weisburg et al., 1991** |
| **bssF1** | ***bssA*** | **GACGARTTCATCRTCGGCTACCACGC** | **Junca. H. Pers. Com.** |
| **bssR2** | ***bssA*** | **AACTCSCTSGTBGCSATCAAGAA** | **Junca. H. Pers. Com** |
| **bssR1** | ***bssA*** | **GTAGCGCTCGATSGCRTGRCAGATCAG** | **Junca. H. Pers. Com** |
| **bcrF** | ***bcrC*** | **CGHATYCCRCGSTCGACCATCG** | **Kuntze et al., 2011** |
| **bcrR** | ***bcrC*** | **CGGATCGGCTGCATCTGGCC** | **Kuntze et al., 2011** |
| **bamASP9F** | ***bamA*** | **CAGTACAAYTCCTACACVACBG** | **Kuntze et al., 2008** |
| **bamA ASP23R**  **M13-F**  **M13-R** | ***bamA*** | **TTTTCCTTGTTGVSRTTCC**  **GTAAAACGACGGCCAGT**  **CAGGAAACAGCTATGAC** | **Kuntze et al., 2008**  **Messing, 1983**  **Messing, 1983** |
